# Exploration of the causal relationship between inflammatory cytokines and type 2 diabetes via mendelian randomization and polygenic score

**DOI:** 10.3389/fimmu.2026.1848697

**Published:** 2026-07-08

**Authors:** Cong Zhang, Yanping Zhao, Hui Pang, Na Li, Haifang Zheng, Jiahui Dong, Yanchao Liu, Wuyun Tana Li, Hailing Li, Nan Zhang, Mengyu Zhao, Lingyan Zhao

**Affiliations:** 1 School of Public Health, Key Laboratory of Molecular Epidemiology of Chronic Diseases, Inner Mongolia Medical University, Hohhot, China; 2 Department of Neurology, The Affiliated Hospital of Inner Mongolia Medical University, Hohhot, China; 3 Hohhot Municipal Center for Disease Control and Prevention (Hohhot Municipal Health Supervision Institute), Hohhot, China; 4 Department of Medical Records and Statistics, Huangshi Central Hospital, Huangshi, China; 5 Institute of Tuberculosis and AIDS Prevention and Control, Inner Mongolia Autonomous Region Center for Disease Control and Prevention, Hohhot, China

**Keywords:** inflammatory cytokines, Mendelian randomization, Mongolian ethnic group, polygenic score, type 2 diabetes

## Abstract

**Background:**

To investigate the potential causal relationship between inflammatory cytokines and type 2 diabetes (T2D) using two-sample Mendelian randomization (MR) and to explore the transferability of inflammatory genetic susceptibility in a Mongolian population through polygenic score (PGS) analysis.

**Methods:**

A two-sample MR analysis was conducted using genome-wide association study (GWAS) summary statistics. Inflammatory cytokine data were obtained from protein quantitative trait locus GWAS datasets (GCST90274758–GCST90274848), and T2D summary statistics were derived from the IEU Open GWAS database (ebi-a-GCST006867). The inverse variance weighted method was used as the primary MR approach, complemented by sensitivity analyses. In an independent Mongolian cohort (N = 351), a PGS was constructed from directly genotyped cytokine-associated SNPs. We assessed PGS-T2D associations using logistic regression adjusted for age, sex, and principal components (PCs), with sensitivity analyses to determine the optimal PC number.

**Results:**

In the European population, genetically predicted higher FGF-21 levels were associated with an increased risk of T2D (OR = 1.141, 95% CI: 1.032–1.261, P = 0.010), and IL-5 also showed a positive association with T2D risk (OR = 1.114, 95% CI: 1.004–1.237, P = 0.042). In contrast, ARTN, CD5, CSF-1, CXCL10, CXCL9, FGF-19, and SLAMF1 were associated with lower T2D risk. In the Mongolian population, the PGS for inflammation-related cytokine levels demonstrated a significant association with T2D status across models adjusting for 0 to 6 principal components (all P< 0.05). In the full model (adjusted for age, sex, and the first 4 PCs), each 1-SD increase in the PGS corresponded to a 25.4% increase in the odds of T2D (OR = 1.254, 95% CI: 1.005–1.575, P = 0.048; AUC = 0.625). These findings are suggestive and exploratory, necessitating further validation in independent cohorts.

**Conclusion:**

This study identified nine inflammatory cytokines with potential causal associations with T2D in European populations. Notably, in a Mongolian cohort, a PGS for genetically predicted cytokine levels showed a modest yet independent association with T2D risk after controlling for population stratification. These cross-population findings warrant replication in larger-scale studies.

## 1 Introduction

Type 2 diabetes (T2D) is a complex metabolic disorder characterized by chronic hyperglycemia resulting from insulin resistance and impaired insulin secretion. According to the International Diabetes Federation, the global number of adults aged 20–79 years living with diabetes reached 589 million in 2024 and is projected to rise to 853 million by 2050 (1). T2D poses a major public health challenge worldwide because of its increasing prevalence and its substantial burden of vascular and metabolic complications.

The Mongolian ethnic group, primarily residing in the Inner Mongolia Autonomous Region of northern China, represents a relatively understudied population in genetic studies of T2D. Their dietary and lifestyle characteristics have often been described as involving low consumption of vegetables and fruits and relatively high intake of meat, dairy fat, salt, and alcohol, which may contribute to the elevated prevalence of chronic metabolic diseases, including T2D (2). In addition to environmental influences, the Mongolian population has a distinct genetic background, making it a potentially informative cohort for evaluating the transferability of genetic findings derived from large-scale studies in other ancestries.

Accumulating evidence indicates that chronic low-grade inflammation plays an important role in the development and progression of T2D. Inflammatory mediators such as interleukin-6 (IL-6) and tumor necrosis factor-α (TNF-α) have been associated with insulin resistance, pancreatic β-cell dysfunction, and broader metabolic dysregulation (3, 4). However, observational studies remain vulnerable to confounding and reverse causation, making it difficult to determine whether inflammatory cytokines are causally involved in T2D pathogenesis.

Mendelian randomization (MR) is a genetic epidemiological approach that uses genetic variants as instrumental variables to infer potential causal relationships between exposures and outcomes (5). Because alleles are randomly allocated at conception, MR can reduce bias from confounding and reverse causality under appropriate assumptions. Previous MR studies have suggested associations between certain inflammatory markers and T2D, but most analyses have been performed in European populations and have focused on a limited number of cytokines (6, 7). In parallel, polygenic score (PGS) analysis provides a complementary framework for evaluating whether aggregated genetic susceptibility identified in one population may show predictive relevance in an independent cohort (8).

In the present study, we first performed a two-sample MR analysis using European GWAS summary statistics to investigate the potential causal effects of circulating inflammatory cytokines on T2D. We then assessed whether part of this inflammation-related genetic susceptibility could be reflected in an independent Mongolian cohort through PGS analysis based on directly genotyped variants. Because genotype imputation did not yield sufficiently reliable data quality in our cohort, we conservatively restricted the PGS analysis to high-quality directly genotyped single nucleotide polymorphisms (SNPs). This design provides complementary evidence by combining causal inference in Europeans with exploratory external validation in an understudied Mongolian population. Our aim was to identify inflammation-related cytokines potentially involved in T2D and to provide a basis for future mechanistic and population-specific studies.

## 2 Methods

### 2.1 Study design and data sources

This study consisted of two components:

1. A two-sample MR analysis conducted using European GWAS summary statistics.
2. A PGS analysis performed in an independent Mongolian case-control population to evaluate the cross-population relevance of inflammation-related genetic susceptibility.

Summary statistics for inflammatory cytokines were obtained from a protein quantitative trait locus GWAS dataset reported by Zhao et al. (GCST90274758–GCST90274848), which included 91 inflammatory cytokines measured in 14,824 individuals of European ancestry (9). Summary statistics for T2D were obtained from the IEU Open GWAS database (ebi-a-GCST006867), based on a large meta-analysis including 62,892 T2D cases and 596,424 controls of European ancestry (10). The Mongolian population comprised 178 T2D cases and 173 controls (N = 351).

### 2.2 Mongolian population

#### 2.2.1 Participant recruitment

Participants were recruited between August 2018 and August 2020 from Tongliao City, Xilingol League, and Hohhot City in the Inner Mongolia Autonomous Region of China. A multistage sampling strategy was used to recruit permanent residents with three consecutive generations of Mongolian ancestry from gacha, villages, and community settings. A total of 2,558 individuals were initially surveyed. Among them, 200 patients with T2D and 200 healthy controls were randomly selected for genotyping. After genotyping quality control, 178 T2D cases and 173 controls remained available for analysis.

The study protocol was approved by the Ethics Committee of Inner Mongolia Medical University (Approval No. YKD201666), and written informed consent was obtained from all participants.

#### 2.2.2 Inclusion and exclusion criteria

##### 2.2.2.1 Inclusion criteria

Case group: 1) Individuals with three generations of direct Mongolian ancestry and no consanguinity; 2) age 18 years or older, male or female; 3) diagnosis of T2D, defined by the presence of diabetes symptoms plus one of the following: random blood glucose ≥ 11.1 mmol/L, fasting plasma glucose ≥ 7.0 mmol/L, 2-hour blood glucose ≥ 11.1 mmol/L during an oral glucose tolerance test, or hemoglobin A1c ≥ 6.5%; and 4) provision of written informed consent and voluntary participation in the study.

Control group: 1) Randomly selected healthy Mongolian individuals; 2) age 18 years or older, male or female; 3) Mongolian ethnicity and no relationship to other study participants; and 4) provision of written informed consent and voluntary participation in the study.

##### 2.2.2.2 Exclusion criteria

1) Pregnant or lactating women; 2) individuals with primary diseases of major organs; 3) individuals who had taken medications affecting glucose or lipid metabolism within the preceding 3 months; and 4) patients with hyperglycemia secondary to other diseases, such as liver disease, endocrine disorders, or severe complications involving the heart, lungs, liver, kidneys, or brain.

#### 2.2.3 Sample collection and genotyping

Five milliliters of fasting venous blood were collected from each participant into ethylenediaminetetraacetic acid anticoagulant tubes and stored at −80 °C until DNA extraction. Genomic DNA was extracted using the Whole Blood Genomic DNA Extraction Kit (Qiagen, Germany). Genotyping was performed by Beijing Letu Medical Laboratory Co., Ltd. using the ASA-CHIA (Infinium Asian Screening Array-China Health Industry Alliance) Bead Chip (Illumina, Inc., San Diego, CA, USA) according to the manufacturer’s standard Infinium HTS assay protocol. The array covered approximately 738,980 genetic variants. All samples underwent standardized quality control, and only samples with acceptable genotyping performance were retained for analysis.

#### 2.2.4 Sample size estimation

Sample size estimation for this case-control study was based on a log-additive genetic model. Referencing a previous study on potassium channel genes (ABCC8) and T2D in the Mongolian population (11), we assumed an effect size (odds ratio, OR) of 1.79 and a control minor allele frequency (MAF) of 0.20. With a two-sided significance level (α) of 0.05, 80% statistical power, and a 1:1 case-to-control ratio, the required total sample size was calculated using the genpwr package (version 1.0.4) in R.

### 2.3 Instrument selection for MR

**Figure 1:**
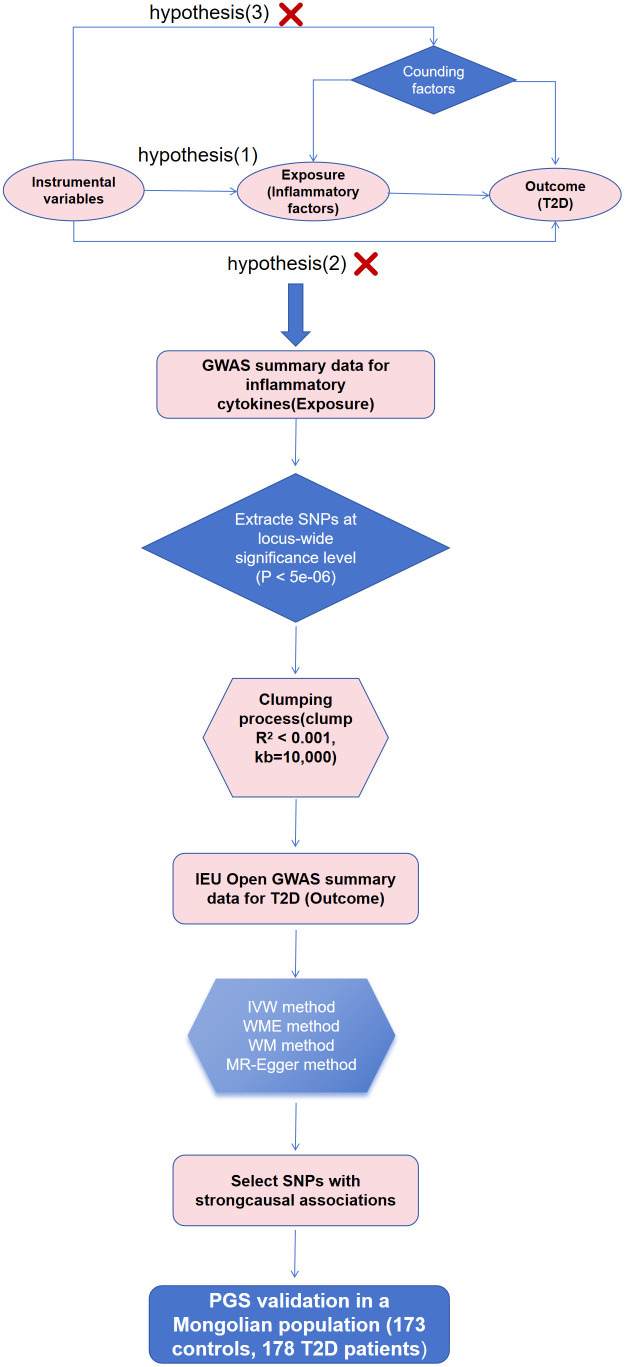
The three fundamental assumptions of MR and the research process.

To assess causal relationships between inflammatory cytokines and T2D, SNPs were used as instrumental variables. Genetic variants significantly associated with inflammatory cytokines were selected as instrumental variables, in accordance with the three core assumptions of MR:

1) Relevance assumption: A genome-wide significance threshold of P< 5 × 10^-8^ was initially applied to identify SNPs strongly correlated with inflammatory cytokines and T2D. However, because only a limited number of SNPs reached this threshold for certain cytokines, a slightly relaxed cutoff (P< 5 × 10^-6^) was adopted to ensure sufficient instrument selection. For cytokines with fewer than three independent SNPs at this threshold (i.e., insufficient instruments for valid Mendelian randomization analysis), a relaxed threshold of P< 5 × 10^-6^ was used to ensure at least three variants were retained (11); 2) Independence assumption: Selected SNPs underwent linkage disequilibrium testing using stringent parameters (R²< 0.001, window size = 10,000 kb) to ensure independence among variants (12); 3) Exclusion restriction assumption: Instrumental variable strength was evaluated using the F-statistic (Formula: F = β²/SE², where β represents the effect on exposure and SE denotes the standard error). SNPs with F< 10 were excluded to minimize weak instrumental variable bias (13). **Figure 1** illustrates these three core assumptions and the overall study workflow. 

### 2.4 MR analysis

The primary MR analysis utilized the inverse variance weighted (IVW) method, supplemented by MR-Egger regression, the weighted median, and the weighted mode methods for validation (14). Heterogeneity among SNP effects was evaluated using Cochran’s Q statistic in the IVW model and Rucker’s Q statistic in the MR-Egger model; P ≥ 0.05 indicated no significant heterogeneity. A random-effects IVW model was applied when heterogeneity was present, whereas a fixed-effects model was used in its absence. Funnel plots were generated for the visual assessment of homogeneity. Potential horizontal pleiotropy was assessed via the MR-Egger intercept test (15, 16) and MR-PRESSO (Mendelian Randomization Pleiotropy Residual Sum and Outlier) analysis. A leave-one-out sensitivity analysis was conducted to evaluate the robustness of the findings and to identify any single SNP exerting a disproportionate influence on the causal estimates (17). Causal estimates were expressed as ORs with 95% CIs.

To account for multiple testing across the 91 circulating inflammatory cytokines analyzed, Bonferroni correction was applied. A two-sided P< 5.5 × 10^-4^ (0.05/91) denoted strong evidence of a statistically significant causal association, whereas associations with nominal significance (P< 0.05 but P ≥ 5.5 × 10^-4^) were considered suggestive evidence (18).

### 2.5 PGS analysis

#### 2.5.1 Genotype quality control and imputation assessment

Quality control of Mongolian genotype data was performed using PLINK v1.9. Samples and SNPs with missing genotype rates > 5% were excluded. SNPs with minor allele frequency< 1% were removed. Hardy-Weinberg equilibrium was evaluated in controls, and SNPs with P< 1 × 10–^6^ were excluded. After quality control, 489,763 SNPs and 351 individuals were retained for downstream analysis.

To improve genomic coverage, genotype imputation was explored using the CKB Imputation online platform of the China National GeneBank DataBase (CNGBdb; https://db.cngb.org/ckb/). This platform uses Beagle 5.2 for phasing and Minimac4 for genotype imputation. The 1000 Genomes Project Phase 3 East Asian reference panel (EAS; n = 504) was used as the reference dataset. Both the reference panel and the target data were aligned to the GRCh37/hg19 genome build.

After imputation, variants were initially filtered according to Minimac4 imputation quality (Rsq > 0.3). A total of 6,437,262 imputed SNPs were obtained, of which 559,529 remained after post-imputation quality control, including filters for missing rate< 5%, Hardy-Weinberg equilibrium P ≥ 1 × 10^-6^, and minor allele frequency ≥ 1%. However, after harmonization with the cytokine-associated loci identified in the European MR analysis, only 6 imputed SNPs were available for PGS construction. Because this limited overlap did not provide a meaningful improvement over the directly genotyped data and could introduce additional uncertainty, the imputed variants were not included in the final PGS analysis.

For the final analysis, we conservatively restricted the PGS to high-quality directly genotyped SNPs that could be harmonized with the MR-derived effect alleles.

#### 2.5.2 Construction of PGS for inflammatory cytokines

To validate the causal relationships identified in the European MR analysis, PGS were constructed for each inflammatory cytokine significantly associated with T2D, using the Mongolian genotyping dataset. Briefly, IVs extracted from the European MR analysis for each cytokine were overlapped with the Mongolian genotyping array; only SNPs present in both datasets were retained. For the retained SNPs, the effect sizes (β_i_), representing the per-allele effect on cytokine levels, were retrieved from the corresponding European GWAS. The PGS for each individual was then calculated under an additive genetic model using the following formula:


$$\text{PGS}=\sum_{i=1}^{n}\left(\text{βi}\times \text{SNPi}\right)$$

where βi represents the effect size of the i-th SNP derived from the MR-based source data, and SNPi represents the number of effect alleles carried by an individual (coded as 0, 1, or 2). The PGS was subsequently standardized to a Z-score for downstream analysis.


$${\text{PGS}}_{\text{std}}=\frac{\text{PGS}-\text{mean}\left(\text{PGS}\right)}{\text{SD}\left(\text{PGS}\right)}$$

#### 2.5.3 Evaluation of population stratification

Principal component analysis (PCA) and the genomic inflation factor (λ) were employed to assess population stratification. Using PLINK software, PCA was performed on genome-wide SNPs pruned for linkage disequilibrium (LD) to derive the top 10 principal components (PCs). The λ value was calculated as the ratio of the median chi-square statistic from a basic association test to the theoretical median of a chi-square distribution with one degree of freedom (0.455). A λ value< 1.05 indicated an acceptable degree of population stratification (19).

#### 2.5.4 Model evaluation and association analysis

Logistic regression models were used to evaluate the association between the PGS and T2D status in the Mongolian population. Two models were assessed:

1. Model 1: adjusted for age, sex, and the first 4 PCs;
2. Model 2: adjusted for age, sex, the first 4 PCs, and the PGS.

The discriminatory ability of each model was assessed using receiver operating characteristic (ROC) curve analysis, and the area under the curve (AUC) was calculated. Paired AUC comparisons between models were performed using the DeLong test.

### 2.6 Statistical software

All statistical analyses were conducted using R software (version 4.3.3) with RStudio (version 2023.09.0 + 463). Two-sample MR analyses were performed using the TwoSampleMR package, with pleiotropy assessed via the MR-PRESSO package. Forest plots were generated using the forestplot package. For the Mongolian population analysis, logistic regression and ROC curve analyses were executed using the pROC package (version 1.18.5) for AUC estimation. Sample size calculation utilized the genpwr package (version 1.0.4). Genotype quality control was performed using PLINK (version 1.9). Statistical significance was defined as a two-sided P< 0.05.

## 3 Results

### 3.1 Baseline data on the Mongolian population

The estimated total sample size required for adequate power (based on a log-additive genetic model, assuming an OR of 1.79, control MAF of 0.20, α=0.05, 80% power, and a 1:1 case-to-control ratio) was 302 (approximately 151 cases and 151 controls), which was surpassed by the actual sample size of 351. **Table 1** presents the baseline data for the Mongolian population. The control group consisted of 173 individuals, while the T2D case group comprised 178 individuals. Analysis of age and gender revealed no statistically significant differences between the two groups (P > 0.05).

**Table 1:** Baseline characteristics of the Mongolian population in PGS analysis.

Characteristic	Total	Cases	Controls	p_value
N	351	178	173	
Age (years), median (IQR)	57.00 (50.00-63.00)	57.00 (50.00-63.00)	57.00 (50.00-63.00)	0.785
Sex, n (%)	351 (100%)	178 (100%)	173 (100%)	1.000
Male	172 (49%)	87 (49%)	85 (49%)	
Female	179 (51%)	91 (51%)	88 (51%)	

N, sample size; median (IQR), continuous variables not following a normal distribution are described using the median (interquartile range).

### 3.2 MR analysis

MR analysis identified associations of fibroblast growth factor 21 (FGF-21), interleukin-5 (IL-5), artemin (ARTN), T-cell surface glycoprotein CD5 (CD5), macrophage colony-stimulating factor 1 (CSF-1), C-X-C motif chemokine 10 (CXCL10), C-X-C motif chemokine 9 (CXCL9), fibroblast growth factor 19 (FGF-19), and signaling lymphocytic activation molecule family member 1 (SLAMF1) with T2D.

**Figure 2:**
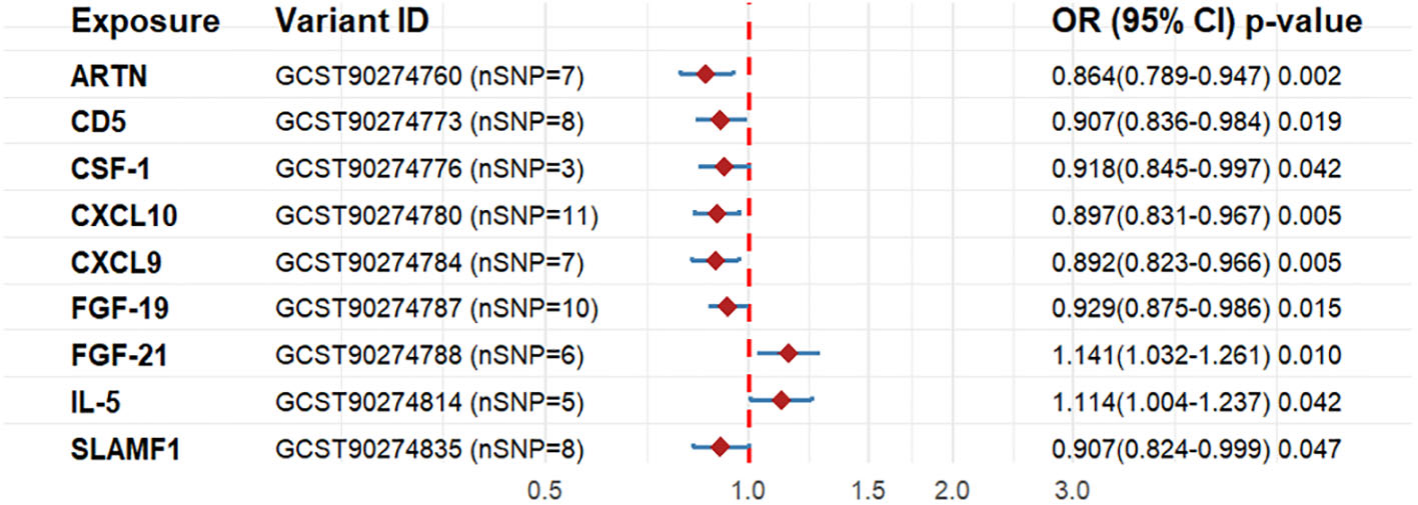
Forest plot showing the causal relationships between 9 inflammatory cytokines and T2D in the IVW results from MR analysis in the European population. P-values shown are raw (unadjusted). The Bonferroni-corrected significance threshold for 91 tests is P< 5.5 × 10^-4^; none of the associations remained significant after correction. These findings are suggestive and require validation in independent cohorts.

**Figure 3:**
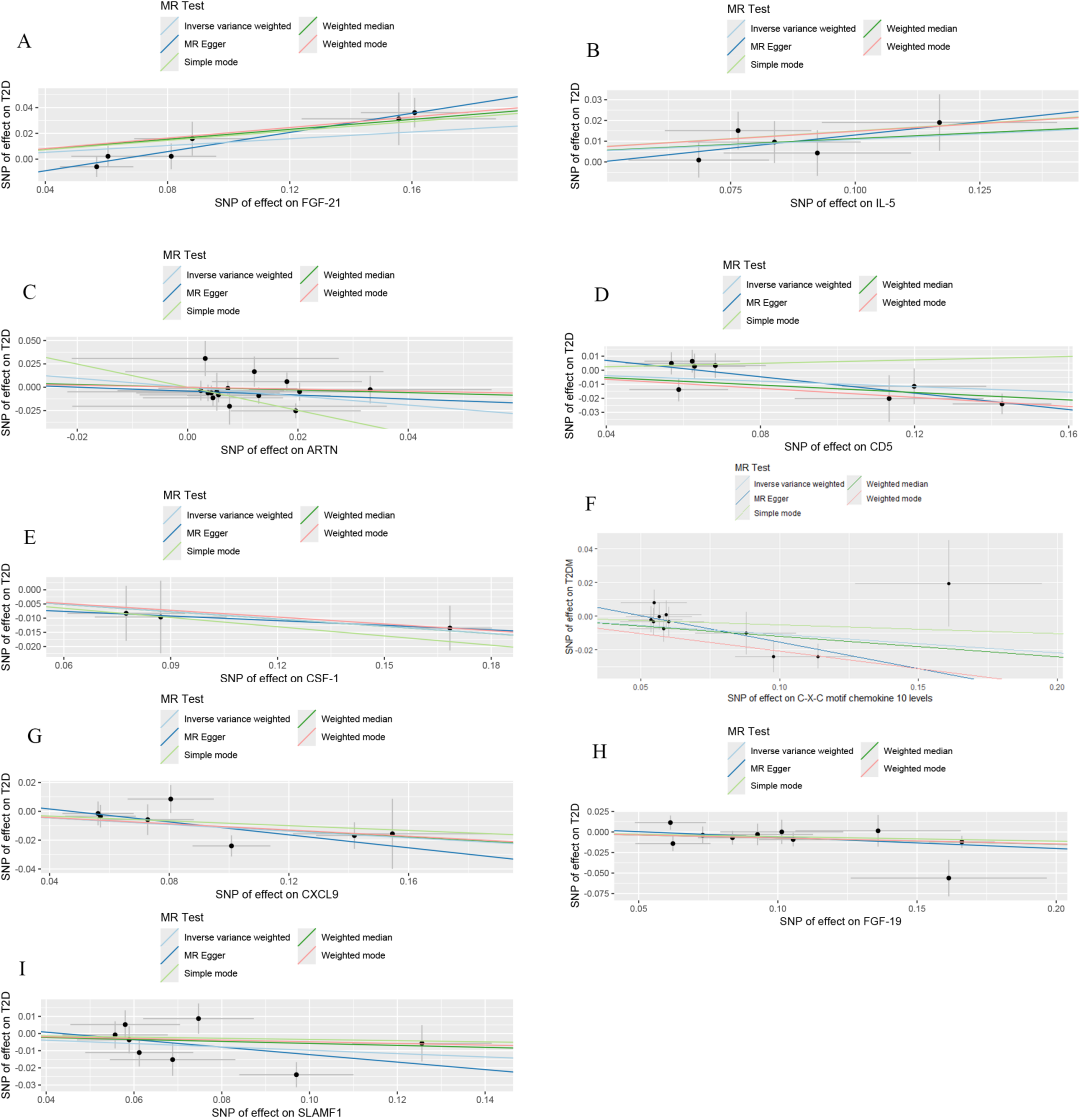
Scatter plot for SNP analysis. **(A)** FGF-21 gene expression; **(B)** IL-5 gene expression; **(C)** ARTN gene expression; **(D)** CD5 gene expression; **(E)** CSF-1 gene expression; **(F)** CXCL10 gene expression; **(G)** CXCL9 gene expression; **(H)** FGF-19 gene expression; **(I)** SLAMF1 gene expression. The x-axis represents the effect of SNPs on inflammatory cytokines, while the y-axis represents the effect of SNPs on T2DM. Each black dot represents an SNP, with lines depicting 95% confidence intervals. The slope of each line reflects the causal estimate derived from the MR method. In this visualization, the light blue line corresponds to the IVW method, the blue line represents the MR Egger method, the dark green line indicates the WME method, and the light red line denotes the WM method.

In the MR analysis of the European population, the IVW method showed that elevated levels of FGF-21 (OR = 1.141; 95% CI = 1.032–1.261, p = 0.01) and IL-5 (OR = 1.114; 95% CI = 1.004–1.237, p = 0.042) were risk factors for T2D; ARTN, CD5, CSF-1, CXCL10, CXCL9, FGF-19, and SLAMF1 demonstrated protective effects. In total, 65 SNPs were extracted from these nine inflammatory cytokines (**Figure 2**). All instrumental variables had F-statistics greater than 10, indicating minimal risk of weak instrumental variable bias and strong model validity (**Supplementary Table 1**). Furthermore, MR scatterplots indicated that in the European cohort, FGF-21 and IL-5 displayed significant positive causal associations with T2D (**Figures 3A, B**), whereas ARTN, CD5, CSF-1, CXCL10, CXCL9, FGF-19, and SLAMF1 showed significant negative causal associations (**Figures 3C–I**). Notably, none of the nominal associations remained significant after applying a Bonferroni correction for 91 tests (P< 5.5 × 10^-4^), indicating that these findings are suggestive rather than definitive and require validation in larger studies.

#### 3.2.1 Sensitivity analysis

**Figure 4:**
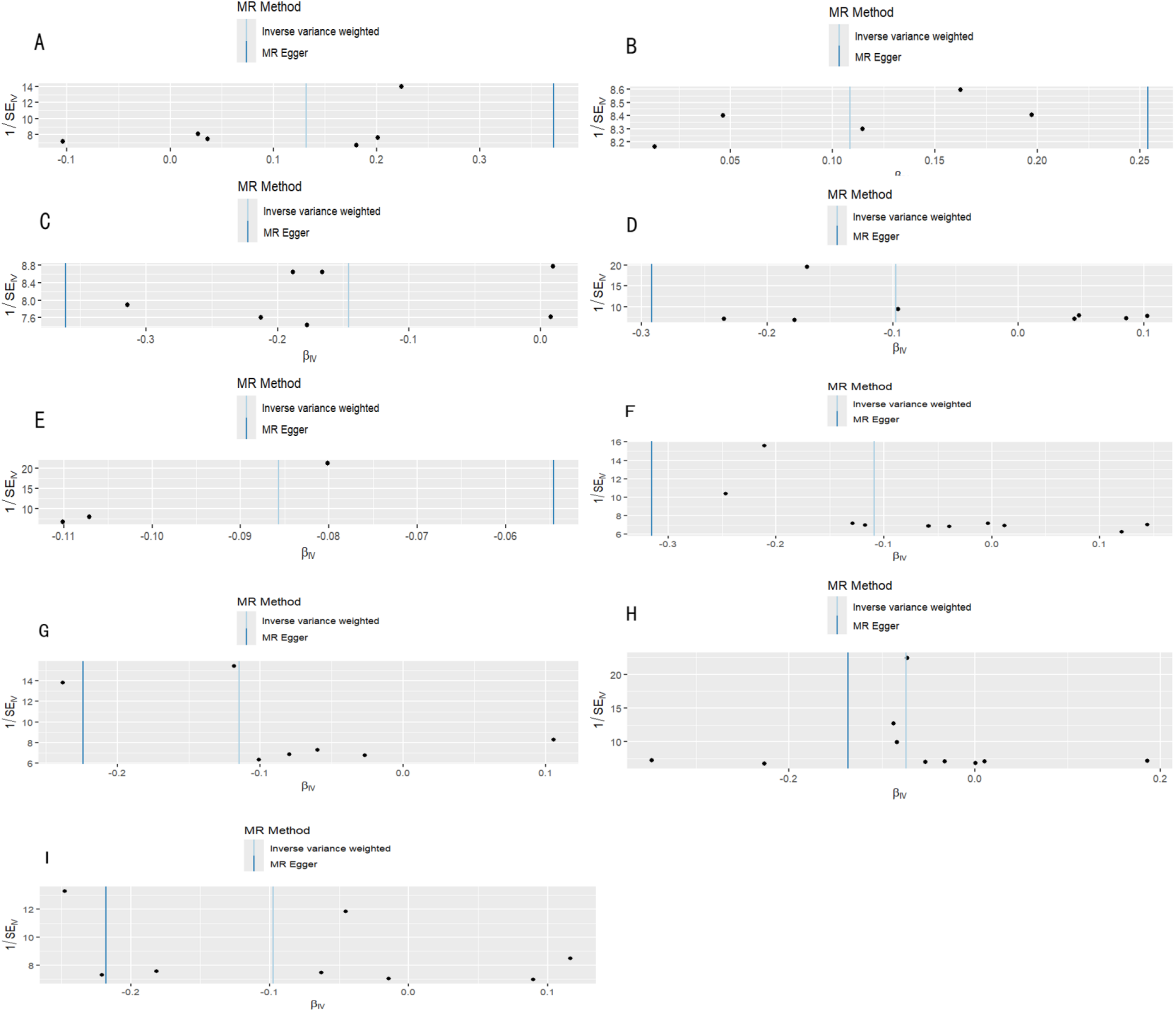
Sensitivity analysis funnel plot. **(A)** FGF-21 gene expression; **(B)** IL-5 gene expression; **(C)** ARTN gene expression; **(D)** CD5 gene expression; **(E)** CSF-1 gene expression; **(F)** Gene expression of CXCL10; **(G)** Gene expression of CXCL9; **(H)** Gene expression of FGF-19; **(I)** Gene expression of SLAMF1. Light blue indicates the IVW method; dark blue indicates the MR Egger method.

**Figure 5:**
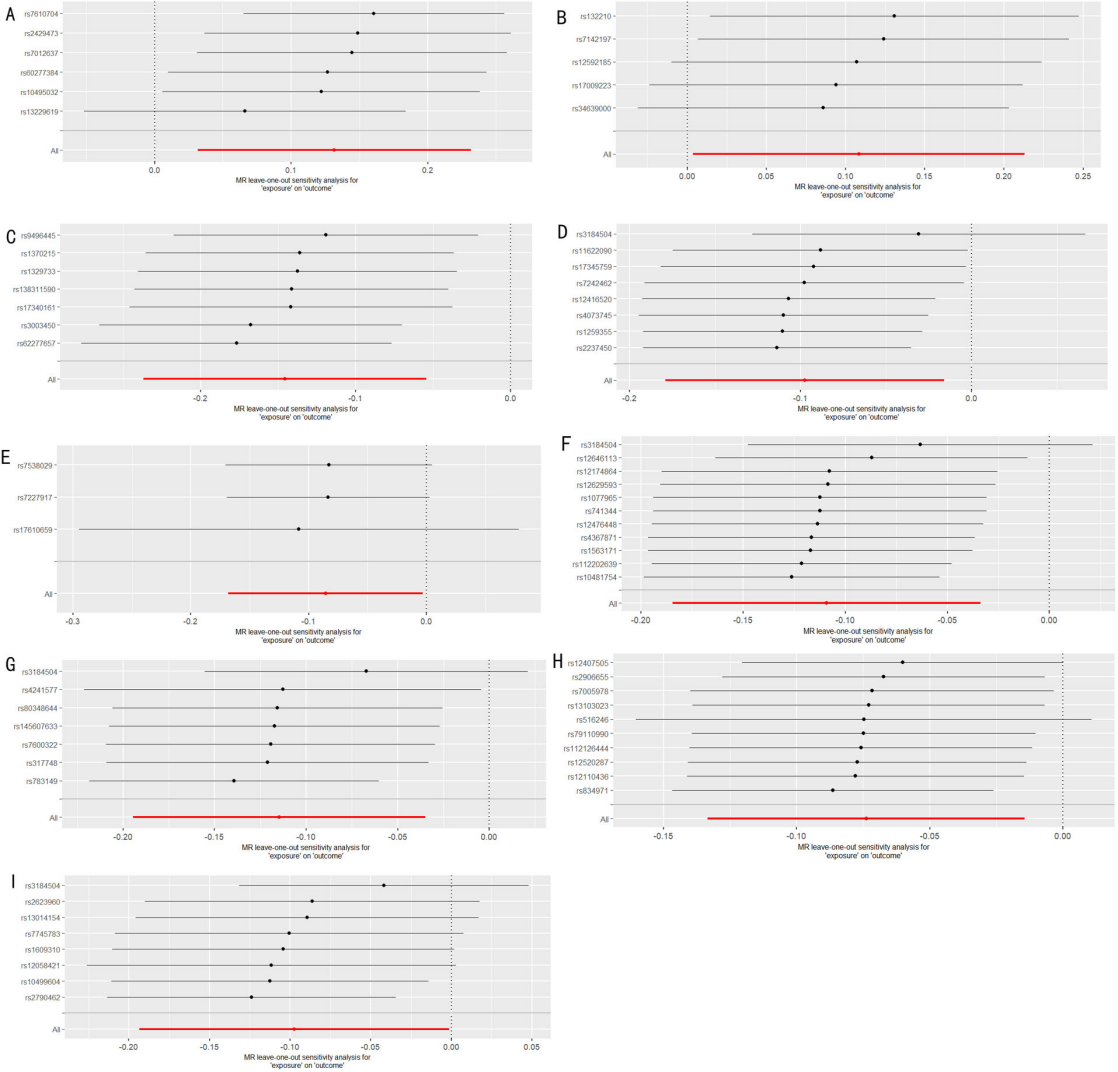
Forest plots of LOO analysis. **(A)** FGF-21 gene expression; **(B)** IL-5 gene expression; **(C)** ARTN gene expression; **(D)** CD5 gene expression; **(E)** CSF-1 gene expression; **(F)** CXCL10 gene expression; **(G)** CXCL9 gene expression; **(H)** FGF-19 gene expression; **(I)** SLAMF1 gene expression. In this plot, each black dot represents the standard deviation (SD) increase in T2D associated with each inflammatory cytokine, estimated using each SNP as an independent instrumental variable (IV). Conversely, red dots denote causal estimates derived from all combined SNPs using various MR methods. Horizontal lines indicate 95% confidence intervals (CI). Specifically, IVW causal estimates illustrate how overall estimates (represented by red horizontal lines) are disproportionately affected by removing individual variants (represented by black horizontal lines).

In the European MR analysis, Cochran’s Q and Rucker’s Q tests indicated no significant heterogeneity among the included instrumental variables (P > 0.05). MR-Egger and MR-PRESSO analyses showed no evidence of directional pleiotropy (P > 0.05) (**Table 2**). Funnel plots showed approximately symmetrical SNP distributions, and leave-one-out analyses indicated that no single SNP materially changed the overall estimates (**Figures 4A–I**, **5A–I**). Together, these findings support the robustness of the MR results.

**Table 2:** Assessment of heterogeneity and pleiotropy.

Source of the crowd	Exposure	Outcome	Heterogeneity test		Pleiotropy test	MR-PRESSO	
			Cochran’s Q test	Rucker’s Q test	Egger intercept	Distortion	Global
			(*P*_value)	(*P*_value)	(*P*_value)	Test	Test
			IVW	MR-egger	MR-egger	Outliers	*P_*value
European population	FGF-21	T2D	0.290	0.935	0.082	NA	0.311
European population	IL-5	T2D	0.799	0.699	0.666	NA	0.819
European population	ARTN	T2D	0.484	0.397	0.603	NA	0.425
European population	CD5	T2D	0.215	0.594	0.068	NA	0.177
European population	CSF-1	T2D	0.965	0.962	0.837	NA	0.685
European population	CXCL10	T2D	0.313	0.548	0.085	NA	0.26
European population	CXCL9	T2D	0.337	0.328	0.385	NA	0.395
European population	FGF-19	T2D	0.417	0.377	0.469	NA	0.505
European population	SLAMF1	T2D	0.139	0.116	0.526	NA	0.148

This table presents the results of sensitivity tests for instrumental variables in the Mendelian randomization analysis. Cochran’s Q test (IVW method) and Rucker’s Q test (MR-Egger method) were used to assess heterogeneity among SNPs. All P-values exceeded 0.05, indicating no significant heterogeneity. The MR-Egger intercept test was applied to examine horizontal pleiotropy. All P-values exceeded 0.05, suggesting no significant horizontal pleiotropy was detected. The MR-PRESSO Global Test yielded non-significant *P*-values, and no outliers were identified, further validating the robustness of the MR analysis results.

### 3.3 PGS analysis

#### 3.3.1 SNPs included in PGS

In total, 65 instrumental SNPs were included in the European MR analysis. Among these, 8 independent directly genotyped SNPs were retained in the Mongolian dataset for PGS construction after matching and deduplication. IL-5 contained one SNP (rs34639000); CD5 contained three SNPs (rs11622090, rs3184504, rs4073745); CSF-1 contained one SNP (rs7227917); CXCL10 contained two SNPs (rs4367871, rs3184504); CXCL9 contained one SNP (rs3184504); FGF-19 contained two SNPs (rs12407505, rs516246); SLAMF1 contained one SNP (rs3184504) (**Supplementary Table 2**). Notably, the rs3184504 locus appeared four times across multiple genes; to avoid redundancy, only one instance was retained, resulting in 8 independent SNPs for subsequent analyses. The distribution of PGS in the Mongolian population is presented in **Supplementary Table 3**.

#### 3.3.2 Assessment of population stratification

**Figure 6:**
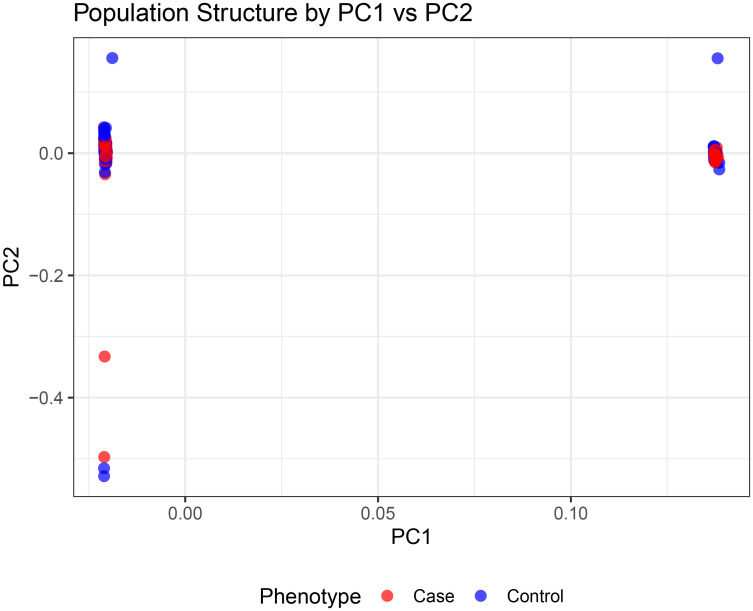
Principal component analysis (PCA) plot of the Mongolian population. PCA was performed using LD-pruned genome-wide SNPs. The first two principal components (PC1 and PC2) are shown. Blue dots represent controls (n = 173) and red dots represent cases (n = 178). PC1 (x-axis) separated the cohort into two genetic clusters (left and right), with cases and controls evenly distributed within each cluster. A few scattered dots represent individuals with intermediate ancestry between the two clusters. The genomic inflation factor (λ = 1.027) confirmed mild population stratification.

Principal component analysis (PCA) revealed two distinct genetic subgroups separated by the first principal component (PC1), indicating the presence of population stratification (**Figure 6**). However, cases (n = 178) and controls (n = 173) were evenly distributed across both subgroups, suggesting that this stratification is unlikely to drive spurious associations between the PGS and T2D. This observation was quantitatively supported by a genomic inflation factor of λ = 1.027 (**Figure 6**), confirming minimal stratification and indicating that the genetic structure of the studied population is relatively homogeneous. Thus, extensive correction for population stratification was deemed unnecessary.

#### 3.3.3 Association between PGS and T2D status

**Figure 7:**
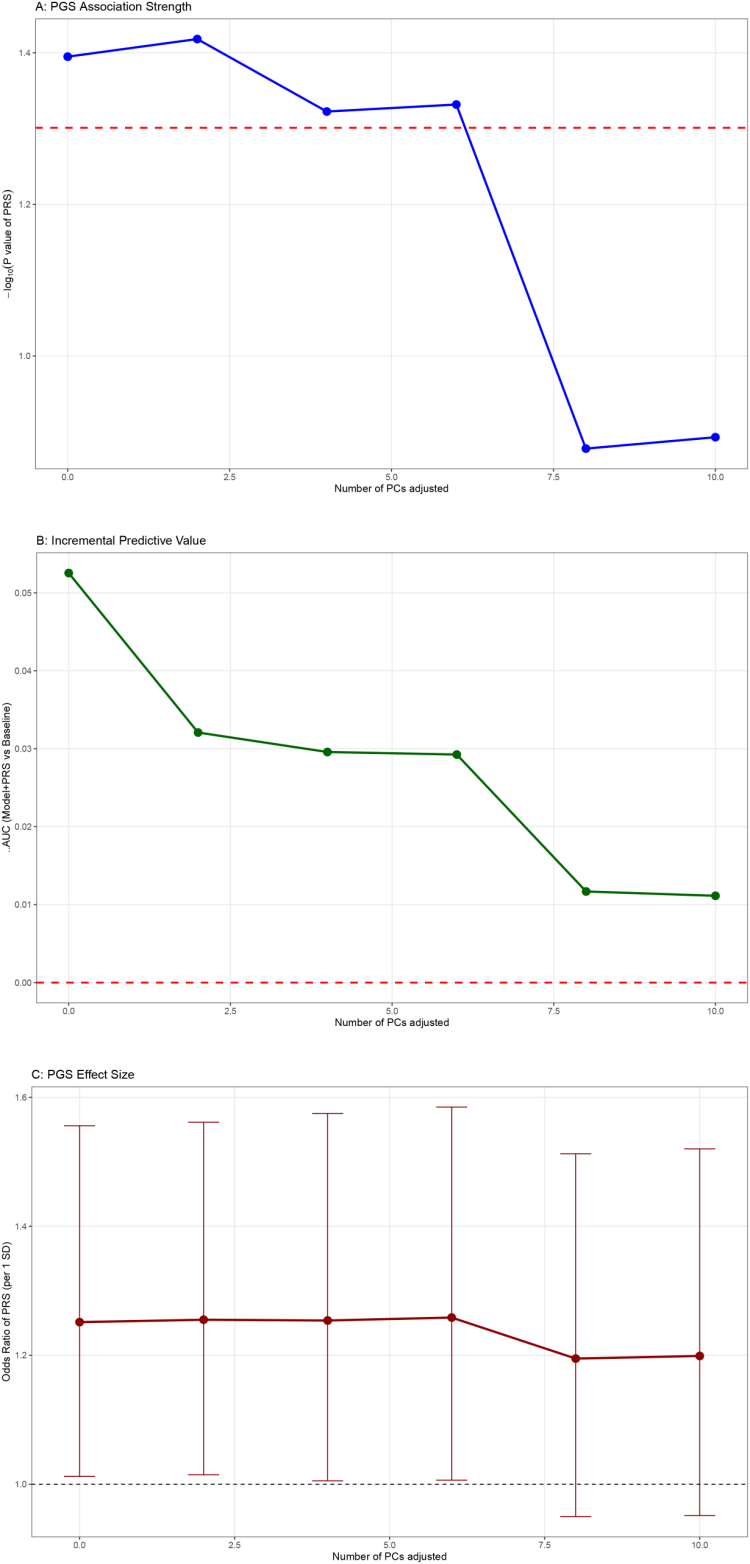
Sensitivity analysis of PGS association with different numbers of PCs. **(A)** -log_10_(P) of PGS; **(B)** ΔAUC upon adding PGS; **(C)** OR (95% CI) of PGS. PGS was significant with 0–6 PCs (all P< 0.05) but non-significant with ≥8 PCs (P > 0.13), indicating over-adjustment. The first 4 PCs were selected as optimal.

A PGS was constructed using eight inflammation-related SNPs, and its association with T2D was evaluated using logistic regression models with stepwise covariate adjustment. To determine the optimal number of PCs for controlling population structure, sensitivity analyses were conducted by adjusting for 0, 2, 4, 6, 8, and 10 PCs (**Table 3**; **Figure 7**). The PGS remained significantly associated with T2D risk when adjusting for 0 to 6 PCs (all P< 0.05), but lost significance upon adjustment for ≥ 8 PCs. Given the modest sample size (N = 351), adjusting for an excessive number of PCs can introduce overfitting and diminish statistical power. Therefore, the first 4 PCs were selected to balance population stratification control and statistical power (**Figure 7**). In the fully adjusted model (Model 2: age, sex, first 4 PCs, and PGS), the PGS demonstrated a significant independent association with T2D (OR = 1.254, 95% CI: 1.005–1.575, P = 0.048). The attenuation of the PGS effect size when adjusting for ≥ 8 PCs likely reflects over-adjustment and power loss in this size-limited cohort, rather than confounding by population stratification.

**Table 3:** Sensitivity analysis of the association between PGS and phenotype under different numbers of adjusted principal components.

n_PC	PGS_OR	95% CI	P value	AUC_baseline	AUC_full	ΔAUC	DeLong P
0	1.25	1.01-1.56	0.04	0.514	0.567	0.053	0.151
2	1.26	1.01-1.56	0.038	0.537	0.57	0.032	0.368
4	1.25	1.01-1.58	0.048	0.596	0.625	0.03	0.112
6	1.26	1.01-1.58	0.047	0.595	0.624	0.029	0.119
8	1.19	0.95-1.51	0.133	0.645	0.657	0.012	0.285
10	1.2	0.95-1.52	0.128	0.647	0.658	0.011	0.315

All models were adjusted for age and sex. The number of principal components (PCs) ranged from 0 to 10. PGS_OR represents the odds ratio associated with a one-standard-deviation increase in the PGS; 95% CI denotes the confidence interval; AUC_baseline is the area under the curve (AUC) of the baseline model (age + sex + the corresponding number of PCs) without PGS; AUC_full is the AUC of the full model including PGS; ΔAUC is the difference between the AUCs of the full model and the baseline model; the DeLong test is used to compare the difference between the two AUCs.

#### 3.3.4 Predictive performance of PGS models

**Figure 8:**
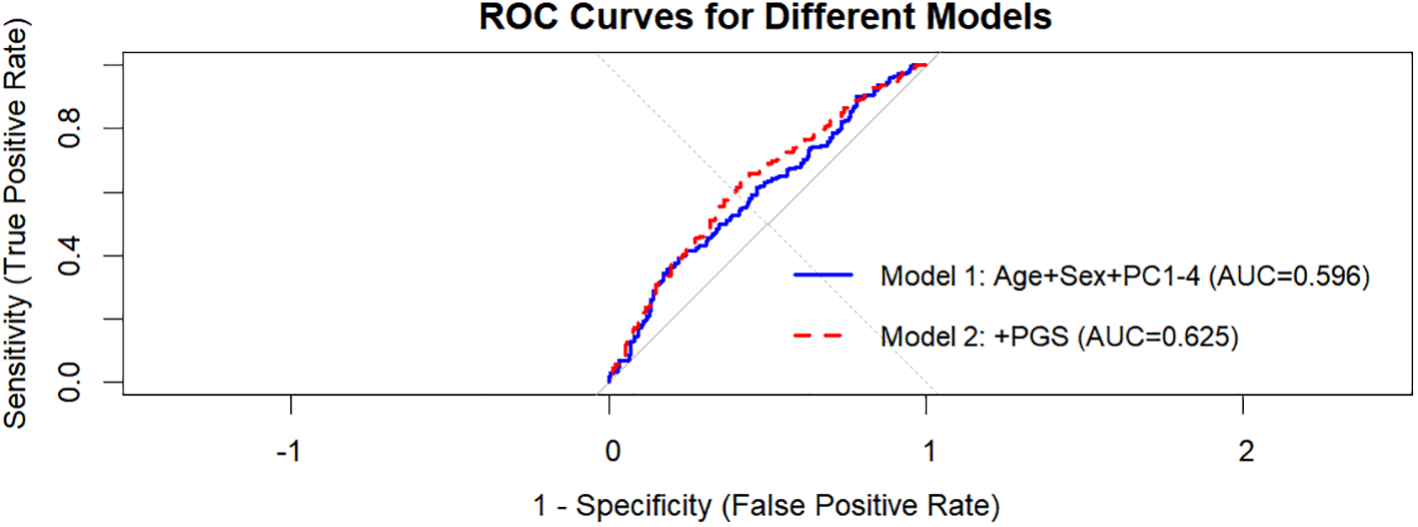
ROC curves of the four logistic regression models. Model 1: age + sex + 4 PCs (AUC = 0.596); Model 2: + PGS (AUC = 0.625). The full model (Model 2) showed the best discriminative performance, with a ΔAUC of 0.029 compared to Model 2 (DeLong test, P = 0.112).

Predictive performance was evaluated for two models using the area under the receiver operating characteristic curve (AUC) (**Figure 8**). Model 1, comprising demographic variables and the first four principal components (PCs), achieved an AUC of 0.596 (95% CI: 0.537–0.655), suggesting that these PCs contribute to disease discrimination. The addition of the PGS to Model 1 (Model 2) increased the AUC to 0.625 (95% CI: 0.566 - 0.684), corresponding to a DAUC of 0.029. However, this incremental value was not statistically significant (DeLong test, P = 0.112) (**Supplementary Table 4**), likely attributable to the limited sample size (N = 351) and the moderate effect size of the PGS (OR = 1.25). Nevertheless, the independent contribution of the PGS is supported by its statistically significant association in Model 2 (P = 0.048). Collectively, these findings suggest a modest but independent association between the inflammation-related PGS and susceptibility to T2D in the Mongolian population.

## 4 Discussion

In this study, we utilized large-scale European GWAS summary statistics to investigate the potential causal effects of inflammatory cytokines on T2D through two-sample MR. We identified nine cytokines associated with T2D in the European analysis. Genetically predicted higher levels of FGF-21 and IL-5 were associated with increased T2D risk, whereas ARTN, CD5, CSF-1, CXCL10, CXCL9, FGF-19, and SLAMF1 showed inverse associations. We further evaluated whether part of this inflammation-related genetic burden could be captured in an independent Mongolian cohort using PGS analysis based on directly genotyped variants. The PGS showed modest discriminatory performance, providing preliminary evidence that some inflammation-related genetic susceptibility may be transferable across populations, although the findings should be interpreted cautiously.

Genetically predicted higher expression levels of FGF-21 and IL-5 were both positively associated with T2D risk. FGF-21 is a multifunctional hormone regulating glucose and lipid metabolism, primarily secreted by the liver and adipose tissue. Under physiological conditions, it exerts significant regulatory effects on glucose and lipid metabolism through mechanisms such as promoting fatty acid β-oxidation, enhancing insulin sensitivity, and suppressing hepatic gluconeogenesis (20–22). However, clinical studies reveal elevated serum FGF-21 levels in diabetic patients, suggesting it may serve as a compensatory marker for metabolic dysfunction under pathological conditions (23). Studies indicate that under acute glucose loading, FGF-21 exhibits a transient elevation followed by a sharp decline, demonstrating a dysregulated dynamic pattern. The magnitude of this fluctuation shows a significant negative correlation with key metabolic parameters like blood glucose and insulin, suggesting impaired acute glucose regulation during stress (24). Combined with previous studies, this acute response impairment, alongside chronic compensatory hyper FGF-21emia triggered by insulin resistance, collectively leads to the failure of its downstream protective mechanism regulating hepatic glucose output by inhibiting the key target glucose-6-phosphatase. Consequently, FGF-21 transforms from a metabolic regulator into a persistent risk factor contributing to the maintenance of hyperglycemic homeostasis (23). Thus, FGF-21 functions as a dual-effect inflammatory mediator: it improves metabolism under physiological conditions, while its elevation in pathological states may reflect compensatory responses to metabolic dysfunction (25). Our findings support a positive correlation between FGF-21 and T2D risk, suggesting its complex pathophysiological role in disease progression.

As a core regulator of eosinophil survival, activation, and recruitment, IL-5 plays a complex and seemingly contradictory dual role in glucose metabolism regulation. Basic research indicates that IL-5 activates the type 2 innate lymphoid cell (ILC2)-eosinophil axis in adipose tissue, driving the polarization of alternative (M2) macrophages and inducing brown fat conversion. This mechanism exerts protective effects in animal models by improving insulin sensitivity and glucose tolerance, whereas IL-5 deficiency or functional inhibition may lead to metabolic deterioration (26, 27). However, clinical studies reveal significantly elevated peripheral blood IL-5 levels in type 2 diabetic patients, with concentrations further increasing in the presence of complications such as diabetic nephropathy (28, 29). In summary, the clinically observed elevation of IL-5 levels in the context of chronic metabolic diseases is more likely to reflect a compensatory or secondary response associated with obesity and low-grade inflammation, rather than a direct pathogenic initiating factor. However, the Mendelian randomization analysis in this study suggests a positive correlation between IL-5 and T2D risk at the genetic level. This provides causal inferential support for the association between the two, indicating that IL-5 may play an active pathophysiological role in disease onset and progression.

We found that multiple factors exhibited protective effects on T2D risk. ARTN, CD5, CSF-1, CXCL9, CXCL10, FGF-19, and SLAMF1all showed negative associations with T2D.

ARTN, a member of the glial cell line–derived neurotrophic factor (GDNF) ligand family and the transforming growth factor beta superfamily, participates in neuroregeneration, pain modulation, and fibrosis via binding to GDNF family receptor alpha 3. Its expression is positively correlated with neuroinflammation severity (30, 31). In pulmonary fibrosis, ARTN exhibits dual functions, acting both as a pro-fibrotic factor and a prognostic biomarker (32) suggesting involvement in the regulation of metabolic inflammation.

CD5 is a glycoprotein expressed on the surface of T and B lymphocytes that helps maintain immune homeostasis by negatively regulating T-cell receptor and B-cell receptor signaling. It is also involved in T helper 17 cell differentiation and interleukin-2 secretion (33, 34), and by facilitating natural IgM production from CD5^+^ B-1a cells to clear metabolic debris and suppress pro-inflammatory macrophage activation (35). CD5 exerts bidirectional immunoregulatory effects in autoimmune and inflammatory diseases (36, 37). Recent studies indicate that CD5 promotes the conversion of autoreactive peripheral CD5hi T cells into extrathymic regulatory T cells (pTregs), independent of its classical role in TCR signaling (37). Mechanistically, CD5 inhibits mTOR activation, counteracting the suppressive effects of certain cytokines (e.g., IL-4, IL-6, IFN-γ) on Treg induction and directly facilitating pTreg generation. This suggests that CD5 may suppress autoimmunity and chronic inflammation in T2D by expanding the pTreg pool (38). Consistent with this, our MR analysis indicates that CD5 confers protection against T2D, likely by maintaining immune balance and metabolic stability. Similarly, CSF-1 (macrophage colony-stimulating factor) may mitigate T2D risk by driving the polarization of tissue macrophages toward an M2-like, metabolically supportive phenotype (characterized by CD206^+^, IL-10^+^, and arginase-1^+^ expression), thereby enhancing insulin sensitivity in adipose tissue and the liver (39, 40). CSF-1 signaling is also indispensable for maintaining this macrophage compartment (41). Collectively, these findings suggest a complementary mechanism wherein CD5 constrains maladaptive T cell activation, while CSF-1 recalibrates innate immunity toward a metabolically permissive state, jointly preserving immune–metabolic homeostasis in T2D.

The inverse associations observed for CXCL9 and CXCL10 were unexpected, given their well-established roles in promoting Th1 polarization and autoimmune inflammation (42–44). Several non-mutually exclusive mechanisms may explain this apparent paradox. First, in the context of T2D, CXCL10-driven macrophage recruitment may preferentially facilitate the infiltration of functionally beneficial macrophage subsets into adipose tissue (43). Second, rather than acting as indiscriminate amplifiers of inflammation, these chemokines may function as context-dependent immunomodulators, potentially biasing macrophage polarization toward an M2-like state (43). Third, their elevated expression in diabetic complications may represent a compensatory response aimed at restraining tissue injury rather than propagating it (44). Finally, beyond their canonical chemotactic functions, CXCL9 and CXCL10 may exert direct metabolic effects on insulin-responsive tissues (45). We therefore hypothesize that CXCL9 and CXCL10 may confer protection against T2D by fine-tuning immune cell trafficking and macrophage polarization in a tissue- and context-specific manner.

FGF-19, a hormone-like protein, regulates bile acid and glucose metabolism through fibroblast growth factor receptor 4, serving as a central mediator of metabolic homeostasis (46). Ileal bile acid absorption activates the farnesoid X receptor to stimulate FGF-19 release, which then suppresses hepatic glucose production and enhances peripheral glucose utilization (47). Clinical evidence supports its metabolic protective properties, including hypoglycemic and hypolipidemic effects (48) consistent with the present MR findings. SLAMF1, a hematopoietic cell–specific immune receptor, appears to negatively regulate inflammatory responses by inhibiting Toll-like receptor–mediated signaling pathways (49, 50). Network MR analysis has suggested that glucagon-like peptide-1 receptor agonists can protect against immunoglobulin A nephropathy via downregulation of SLAMF1 expression (51), consistent with our association findings.

A further strength of this study is the inclusion of a Mongolian cohort, an ethnic group that remains markedly underrepresented in genetic studies of T2D. Rather than employing this cohort for causal inference, we leveraged it as an external validation set to examine whether inflammation-related genetic susceptibility identified in European populations translates to a genetically distinct ancestry. This methodological distinction is crucial: the MR analyses relied exclusively on European GWAS summary statistics, whereas the Mongolian cohort served solely for PGS-based validation. In this external dataset, the PGS was significantly associated with T2D status in baseline and partially adjusted models, although the effect size attenuated upon further adjustment for principal components (PCs). Notably, the fully adjusted model yielded the highest AUC, implying that ancestry-related genetic structure contributed meaningfully to case–control discrimination.

The modest predictive performance of the PGS in the Mongolian cohort is unsurprising, given the limited sample size and the restricted number of directly genotyped variants available for score construction. Although the European MR analysis identified a broader set of cytokine-associated loci, only a small fraction overlapped with the Mongolian genotyping array. We therefore explored genotype imputation using an East Asian reference panel to expand variant coverage; however, following stringent post-imputation quality control and harmonization, only six imputed SNPs remained eligible for inclusion. Given that this marginal gain in coverage was unlikely to materially improve predictive performance and could introduce additional uncertainty, we conservatively restricted the final PGS to directly genotyped SNPs that passed quality control and allele harmonization. Although this strategy reduced the number of variants in the score and likely attenuated its predictive accuracy, it enhanced the robustness, transparency, and interpretability of our findings.

The inclusion of both positively and inversely associated loci in the PGS was methodologically reasonable. PGS reflects the aggregate weighted contribution of multiple variants, including both risk-increasing and risk-decreasing alleles (52). In addition, because the MR estimates were derived from European data and the PGS was evaluated in a Mongolian cohort, the magnitude of individual effects might vary across populations. Previous studies have demonstrated that polygenic scores often show reduced portability when transferred from European to non-European populations due to differences in linkage disequilibrium patterns, allele frequencies, and genetic architecture (53). Retaining all eligible inflammation-related loci therefore allowed a broader assessment of the cumulative contribution of this pathway to T2D susceptibility.

This study has several strengths. First, the MR component leveraged large-scale GWAS data and multiple sensitivity analyses, improving the robustness of causal inference at the genetic epidemiological level. Second, the study extends prior work by systematically evaluating a broad panel of inflammatory cytokines rather than focusing on only a few canonical markers. Third, the use of an independent Mongolian cohort provides preliminary cross-population evidence in an understudied ethnic group and highlights both the opportunities and challenges of transferring genetic findings across ancestries.

Several limitations should be acknowledged. First, regarding generalizability and statistical inference, the MR analysis was conducted exclusively in European populations; thus, the causal estimates may lack direct trans-ethnic generalizability to Mongolian or other non-European groups. Furthermore, after stringent Bonferroni correction for multiple comparisons (9 cytokines; corrected threshold P< 0.0056), no associations retained statistical significance. Consequently, the findings based on nominal uncorrected P-values should be interpreted as exploratory and hypothesis-generating.

Second, statistical power was a notable constraint. Although sample size calculations (based on an assumed OR of 1.79 and MAF of 0.20) indicated adequate power for the Mongolian cohort (N = 351), the study remained underpowered to detect modest genetic effects or small improvements in discriminatory accuracy (e.g., observed ΔAUC = 0.029 for the PGS in the full model). Similarly, the limited sample size precluded more fine-grained adjustment for population structure beyond the first 4 PCs.

Third, the construction and validation of the PGS were limited by genetic data constraints. The overlap between MR-derived loci and the Mongolian genotyping array was restricted, and genotype imputation failed to meaningfully improve this coverage. Consequently, the PGS was based on only 8 directly genotyped SNPs, which cannot fully capture the polygenic architecture of inflammation-related T2D risk. Additionally, a relaxed instrument selection threshold (P< 5 × 10^-6^) was adopted for certain cytokines. Although all retained instruments exhibited F-statistics > 10 and sensitivity analyses supported causal consistency, the potential for weak instrument bias cannot be entirely excluded.

Finally, methodological and interpretive caveats apply. The conventional linearity assumption of MR may not capture potential nonlinear, threshold, or U-shaped relationships between cytokine levels and T2D risk. Moreover, as genetic epidemiological analyses, our findings infer causality but do not elucidate underlying molecular mechanisms. Accordingly, the modest predictive performance of the PGS indicates that it is not yet suitable for standalone clinical application. Future validation in larger, multi-ethnic cohorts, coupled with functional and multi-omics investigations, is essential to confirm these findings and unravel the underlying biology.

## 5 Conclusion

In conclusion, this study identified several inflammatory cytokines with potential causal associations with T2D in European populations using two-sample MR. Genetically predicted higher levels of FGF-21 and IL-5 were associated with increased T2D risk, whereas ARTN, CD5, CSF-1, CXCL10, CXCL9, FGF-19, and SLAMF1 showed inverse associations. In an independent Mongolian cohort, a PGS constructed from directly genotyped cytokine-related variants showed modest but significant independent association, providing preliminary evidence that inflammation-related genetic susceptibility may contribute to T2D risk across populations. These findings help prioritize candidate cytokines for future mechanistic investigation, while larger ancestry-diverse studies and functional validation are required to confirm their biological and clinical significance.

## Data Availability

The inflammatory cytokine datasets GSCT90274758–GSCT90274848 for this study can be found in the GWAS database https://www.ebi.ac.uk/gwas/. The type 2 diabetes datasets GCST006867 for this study can be found in the IEU Open GWAS https://opengwas.io/datasets/. Research findings on the Mongolian population’s gene chip data presented in the study are included in the article/**Supplementary Material**. Due to the sensitive nature of the data (e.g., patient information, ethnic issues), access is restricted. Data are available from the corresponding author upon reasonable request and with permission from the Inner Mongolia Medical University Medical Ethics Committee.

## Supplementary material

Supplementary Table 1 F-statistics for Instrumental Variables.

Supplementary Table 2 SNP Information Successfully Matched in the Mongolian Population.

Supplementary Table 3 Distribution of PGS Scores in the Mongolian Population.

Supplementary Table 4 Predictive performance of the four logistic regression models.
